# A population-based study of tumor gene expression and risk of breast cancer death among lymph node-negative patients

**DOI:** 10.1186/bcr1412

**Published:** 2006-05-31

**Authors:** Laurel A Habel, Steven Shak, Marlena K Jacobs, Angela Capra, Claire Alexander, Mylan Pho, Joffre Baker, Michael Walker, Drew Watson, James Hackett, Noelle T Blick, Deborah Greenberg, Louis Fehrenbacher, Bryan Langholz, Charles P Quesenberry

**Affiliations:** 1 Division of Research, Kaiser Permanente, Oakland, California, USA; 2 Genomic Health, Inc., Redwood City, California, USA; 3 Permanente Medical Group Regional Laboratory, Kaiser Permanente, Berkeley, California, USA; 4 Oncology, Kaiser Permanente, Vallejo, California, USA; 5 USC Keck School of Medicine, Los Angeles, California, USA

## Abstract

**Introduction:**

The Oncotype DX assay was recently reported to predict risk for distant recurrence among a clinical trial population of tamoxifen-treated patients with lymph node-negative, estrogen receptor (ER)-positive breast cancer. To confirm and extend these findings, we evaluated the performance of this 21-gene assay among node-negative patients from a community hospital setting.

**Methods:**

A case-control study was conducted among 4,964 Kaiser Permanente patients diagnosed with node-negative invasive breast cancer from 1985 to 1994 and not treated with adjuvant chemotherapy. Cases (*n *= 220) were patients who died from breast cancer. Controls (*n *= 570) were breast cancer patients who were individually matched to cases with respect to age, race, adjuvant tamoxifen, medical facility and diagnosis year, and were alive at the date of death of their matched case. Using an RT-PCR assay, archived tumor tissues were analyzed for expression levels of 16 cancer-related and five reference genes, and a summary risk score (the Recurrence Score) was calculated for each patient. Conditional logistic regression methods were used to estimate the association between risk of breast cancer death and Recurrence Score.

**Results:**

After adjusting for tumor size and grade, the Recurrence Score was associated with risk of breast cancer death in ER-positive, tamoxifen-treated and -untreated patients (*P *= 0.003 and *P *= 0.03, respectively). At 10 years, the risks for breast cancer death in ER-positive, tamoxifen-treated patients were 2.8% (95% confidence interval [CI] 1.7–3.9%), 10.7% (95% CI 6.3–14.9%), and 15.5% (95% CI 7.6–22.8%) for those in the low, intermediate and high risk Recurrence Score groups, respectively. They were 6.2% (95% CI 4.5–7.9%), 17.8% (95% CI 11.8–23.3%), and 19.9% (95% CI 14.2–25.2%) for ER-positive patients not treated with tamoxifen. In both the tamoxifen-treated and -untreated groups, approximately 50% of patients had low risk Recurrence Score values.

**Conclusion:**

In this large, population-based study of lymph node-negative patients not treated with chemotherapy, the Recurrence Score was strongly associated with risk of breast cancer death among ER-positive, tamoxifen-treated and -untreated patients.

## Introduction

Approximately 65% of women currently diagnosed with invasive breast cancer have lymph node-negative disease at diagnosis [1], and 85% of these women are expected to be alive and free from distant metastasis at 10 years [2]. Chemotherapy in this group of patients, especially among patients with estrogen receptor (ER)-positive disease treated with adjuvant hormonal therapy, offers only a modest improvement in 10-year survival [2,3]. Nonetheless, according to current guidelines the majority of node-negative breast cancer patients should be considered for chemotherapy [4-6]. This is largely because of our limited ability to identify those individual patients who are unlikely to benefit from such treatment.

Multigene assays may provide information on patient prognosis and response to therapy that is superior and/or complementary to that from standard histopathological and immunohistochemical techniques [7,8]. However, multiple well conducted and controlled observational studies or clinical trials in independent populations are needed to establish the clinical usefulness of these assays [9,10].

**Figure 1:**
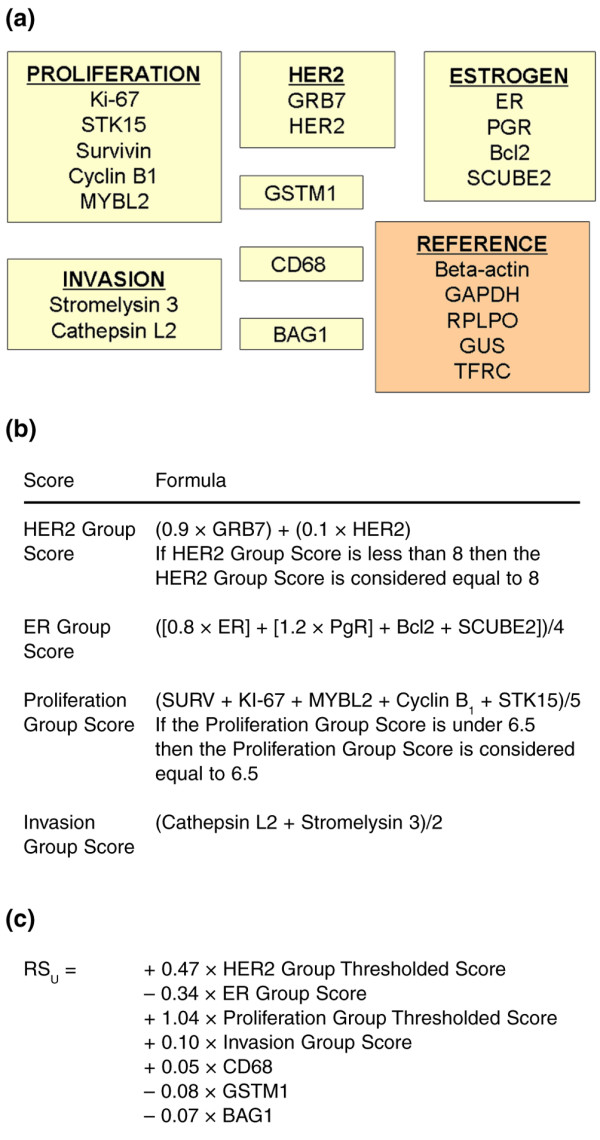
Twenty-one gene panel and calculation of recurrence score. **(a) **The final gene list (16 cancer-related and five reference genes) and summary score (Recurrence Score) algorithm for this assay were developed by analyzing the results of three independent preliminary breast cancer studies (for instance, training sets) with a total of 447 patients [11]. The Recurrence Score, on a scale from 0 to 100, is derived from the reference-normalized expression measurements in four steps. In the first step the expression for each gene is normalized relative to the expression of the five reference genes (b-actin, GAPDH, GUS, RPLPO, and TFRC). Reference-normalized expression measurements range from 0 to 15, where a 1-unit increase reflects approximately a twofold increase in RNA. **(b) **In the second step the HER2 Group Score, the ER Group Score, the Proliferation Group Score, and the Invasion Group Score are calculated from individual gene expression measurements. **(c) **In the third step the Recurrence Score unscaled (RS_U_) is calculated using coefficients that were pre-defined based on regression analysis of gene expression and recurrence in the three training studies (Providence, Rush, and NSABP B-20 [12]). A plus sign indicates increased expression is associated with increased recurrence risk. A minus sign indicates that increased expression is associated with decreased recurrence risk.

Using a multistep approach, a 21-gene assay (Oncotype DX) was recently developed for use in paraffin-embedded tumor tissue to predict risk for distant recurrence or death in lymph node-negative breast cancer patients [11]. Approximately 250 genes, selected from the published literature, genomic databases, pathway analysis, and from microarray-based gene expression profiling experiments performed using fresh frozen tissue, were considered as candidates. The final gene list (16 cancer-related and five reference genes) and summary score (Recurrence Score) algorithm for this assay (Figure 1) were developed by analyzing the results of three independent preliminary breast cancer studies (that is, training sets) conducted in a total of 447 patients [12].

Two subsequent studies were conducted to evaluate the performance of the Recurrence Score in predicting distant recurrence-free survival in lymph node-negative breast cancer patients not treated with chemotherapy. Among 668 tamoxifen-treated patients in the National Surgical Adjuvant Breast and Bowel Project (NSABP) B-14 clinical trial [12], the Recurrence Score was strongly predictive of risk for distant recurrence, both alone and in multivariate analyses that also included patient age, tumor size, tumor grade, and ER and HER2 status. For patients categorized as low, intermediate, or high risk based on Recurrence Score, the Kaplan-Meier estimates for the rates of distant recurrence at 10 years (and the associated 95% confidence intervals [CIs]) were 6.8% (95% CI 4.0–9.6%), 14.3% (95% CI 8.3–20.3%), and 30.5% (95% CI 23.6–37.4%), respectively. A second study [13] evaluated the assay among 149 patients not treated with adjuvant therapy (hormonal or chemotherapy) at the MD Anderson Cancer Center; it found no clear association between the Recurrence Score and risk for distant recurrence. Rates of distant recurrence at 10 years were 18% (95% CI 7–30%), 38% (95% CI 15–61%), and 28% (95% CI 13–32%) for the low, intermediate, and high risk categories, respectively.

To evaluate the Oncotype DX assay in a third independent study population of lymph node-negative breast cancer patients not treated with chemotherapy, we conducted a case-control study among a large population of women who were diagnosed and treated at 14 hospitals and clinics within the Northern California Kaiser Permanente health plan. A pre-specified primary aim of the study was to assess the degree to which the Recurrence Score would predict the risk of breast cancer-specific mortality among women with ER-positive, node-negative breast cancer treated with tamoxifen (that is, patients clinically similar to those included in the study conducted by Paik and coworkers [12]), either alone or in multivariate analyses with tumor size and tumor grade. A pre-specified secondary aim was to examine the extent to which the Recurrence Score predicts risk of breast cancer-specific mortality among ER-positive, node-negative patients not treated with tamoxifen. A pre-specified exploratory aim was to examine the association between risk of breast cancer death and Recurrence Score among node-negative, ER-negative patients.

## Materials and methods

### Study population and design

We conducted a case-control study nested within a cohort of breast cancer patients. The Northern California Kaiser Permanente tumor registry, a contributor to the Surveillance, Epidemiology, and End Results (SEER) program of cancer registries, was used to identify all female health plan members who were diagnosed with lymph node-negative invasive breast cancer from 1985 to 1994. Northern California Kaiser Permanente is a nonprofit, integrated health services delivery organization that provides care for over 3 million members at 14 Northern California Kaiser hospitals and 23 outpatient clinics. The Kaiser Permanente membership is racially and ethnically diverse and is demographically similar to the general population of northern California, although it tends to under-represent the extremes of the socioeconomic spectrum [14-16]. The study was approved by the Kaiser Permanente Institutional Review Board.

Breast cancer patients were eligible if their nodal status was documented to be negative by pathologic assessment, they were diagnosed before age 75 years, and their initial disease was not treated with chemotherapy. Patients were excluded for the following reasons: inflammatory or bilateral breast cancer or evidence of metastasis (including lymph nodes) at initial diagnosis; prior invasive cancer (breast or other) at diagnosis; or unknown/unconfirmed treatment with tamoxifen.

Using the Kaiser Permanente tumor registry and electronic membership databases, patients were followed until death due to breast cancer, death from another cause, bilateral breast cancer, termination of membership, or December 2002, whichever came first.

Cases were patients whose first event was death from breast cancer. At each case's death, up to three controls were randomly selected from patients alive and under follow up (that is, incidence density sampling) [17]. Controls were individually matched to their case with respect to age (within 1 year), race (non-Hispanic white, Hispanic, Black, Asian), calendar year of diagnosis (exact year), Kaiser Permanente pathology department of origin, and treatment of index breast cancer with tamoxifen (yes, no). Note that matching on tamoxifen treatment maximized our ability to conduct analyses stratified by tamoxifen treatment. Not matching on tumor size or tumor grade allowed us to examine whether these measures provide information on risk of breast cancer death that is independent of Recurrence Score. Matching requirements were relaxed to find up to three eligible controls per case. Matching criteria were relaxed in the following order: age was relaxed to < 50 versus ≥ 50 years; calendar year of diagnosis was relaxed to ± 1 year; calendar year of diagnosis was relaxed to ± 2 years; and pathology facility was dropped as a matching factor.

The medical records of cases and controls were reviewed to confirm the initial diagnosis, treatment and cause of death, and to obtain information on eligibility criteria. Pathologic tumor size was obtained from pathology reports. Information on ER and progesterone receptor (PgR) status of the index tumor (usually assessed by the ligand-binding assay) was also abstracted from the medical record.

Cases and controls were selected and eligibility was determined before laboratory measurement of gene expression. Of the 402 cases identified as potentially eligible by the tumor registry, 269 were determined to be eligible by chart review. The eligibility of 27 cases could not be determined because of incomplete or unavailable medical records and were considered lost to the study. Similarly, 722 of the 989 controls initially matched to eligible cases were determined to be eligible by chart review and the eligibility of 29 could not be determined. Of those eligible by chart review, 31 cases and 91 controls were lost because of missing tumor blocks. Another four cases were lost because we were unable to find at least one matched control. This left 234 cases and 631 controls available for pathology studies.

### Blinding and batching of pathology and laboratory procedures

All pathology and laboratory procedures (slide review, sectioning of tumor blocks, macro-dissection, RT-PCR assays, and Recurrence Score calculations) were conducted blinded to the case-control status of patient specimens. In addition, all batches of pathology materials sent to Kaiser Permanente Regional Laboratory, NSABP Pathology, and Genomic Health, Inc. included a mixture of cases and controls.

### Sample preparation

For eligible cases and controls, the hematoxylin and eosin stained slides from biopsies and/or surgeries performed at the time of the index diagnosis were reviewed using pre-specified criteria in order to identify the most representative block and to evaluate whether sufficient tumor tissue was present. Specimens with no tumor or very little tumor (<5% of the area occupied by invasive cancer cells compared to the area occupied by other epithelial elements, such as normal epithelium, fibrocystic change, or ductal or lobular carcinoma *in situ*) were excluded from the study (*n *= 18). Specimens with regions on the slide having prominent nontumor elements (such as smooth muscle, hemorrhage, fibrosis, hyperplastic epithelium, and/or normal breast; but not ductal or lobular carcinoma *in situ *or necrosis) where the nontumor elements were both sufficiently localized to be amenable to macro-dissection and sufficiently abundant (>50% of the overall tissue on the slide) were sent to NSABP Pathology for macro-dissection (*n *= 59). Macro-dissection to obtain enriched tumor tissue was performed using a safety blade cleaned with RNaseZAP (Ambion, Austin, TX, USA) by NSABP Pathology on six (10 μm) sections of the region enriched in tumor tissue. For all other specimens, three (10 μm) sections were prepared by Kaiser Permanente Regional Laboratory, placed into a microcentrifuge tube, and sent to Genomic Health, Inc.

### Tumor size, grade, and estrogen receptor status

When not recorded on the pathology report (7% of reports), tumor size was obtained from a pathology review of all hematoxylin and eosin stained slides from all surgeries at diagnosis. Tumor grade, based on review of an hematoxylin and eosin slide from the most representative block, was assessed independently by two board certified (in anatomic and clinical pathology), assistant professors in departments of pathology, using the modified Bloom-Richardson grading criteria [18]. ER status of the index tumor was unavailable in the 10- to 20-year-old medical record for a significant proportion of patients (16%), and therefore all tumors were classified as positive or negative based on ER expression by RT-PCR (>6.5 and ≤6.5, respectively; cutoff points based on prior studies examining correlation of RT-PCR with immunohistochemistry) [11,13,19]. In our study population, the concordance of ER status from RT-PCR versus from medical chart information was moderate (kappa 0.49, 95% CI 0.41–0.56). All but seven (out of a total of 122) of the discordances were patients classified as ER positive based on RT-PCR and ER negative based on information in the medical chart.

### RT-PCR assay of gene expression and calculation of the Recurrence Score

Gene expression analysis in fixed paraffin-embedded tumor tissue was performed by Genomic Health, Inc., as described previously [11,12]. Briefly, after RNA extraction and DNase I treatment, total RNA content was measured and the absence of DNA contamination was verified. Reverse transcription was performed followed by quantitative RT-PCR reactions in 384 well plates using Applied Biosystems PRISM^® ^7900 HT instruments (Applied Biosystems, Foster City, CA, USA).

Expression of each gene was measured in triplicate wells, and then normalized relative to a set of five reference genes (β-actin, GAPDH, GUS, RPLPO, and TFRC). Reference-normalized expression measurements range from 0 to 15, where a 1-unit increase reflects approximately a twofold increase in RNA.

The potential impact of tumor heterogeneity on Recurrence Score results was examined in two small studies (unpublished data). In a study of 20 patients and 60 blocks (two to five blocks/patient), analysis of variance was used to examine the variability of Recurrence Score between blocks from the same patient (tissue sections did not undergo macro-dissection). The standard deviation in Recurrence Scores (as a continuous value) between blocks was 3.0 Recurrence Score units. For 16 of the 20 patients, the standard deviation between blocks was less than 2.5 Recurrence Score units. A similarly high concordance (Pearson's *r *= 0.86) was also observed between Recurrence Score results from core biopsies and resections among 49 patients with locally advanced breast cancer.

Recurrence Score cutoff points were pre-specified and classified patients into low risk (Recurrence Score <18), intermediate risk (Recurrence Score 18–30) and high risk (Recurrence Score ≥31) categories. All methods used for measurement of gene expression levels and for the calculation of Recurrence Score and gene group scores were identical to those used in the study conducted by Paik and coworkers [12].

### Statistical analysis

Statistical analyses were conducted entirely by Kaiser Permanente researchers following a pre-specified plan. As specified, results were generated for patient groups, based on ER status and tamoxifen treatment.

The Recurrence Score was examined as a continuous variable in 50-unit increments, for consistency and comparison with previously published Recurrence Score findings. In additional analyses, women were categorized into presumptive low, intermediate, and high risk groups based on cutoff points (Recurrence Score < 18, 18–30 and ≥31, respectively), and when categorized according to Recurrence Score quartiles.

When examining the relationships between risk of breast cancer death and the expression level of individual genes, the expression levels were treated as continuous variables. When examining gene groups, the proliferation gene group and the HER2 gene group scores were treated as continuous variables both with and without transformation based on a threshold value (see the Recurrence Score algorithm in Figure 1).

Tumor size was examined both as a continuous variable in 2 cm units (for consistency with the NSABP B-14 study [12]) and when categorized as ≤1 cm, 1.1–2 cm, 2.1–4 cm, and >4 cm. Tumor grade was examined as a categorical variable (well differentiated, moderately differentiated, and poorly differentiated). The grading used was from the standardized re-review. We examined tumor grade separately for pathologist 1 and pathologist 2.

Conditional logistic regression was used to calculate odds ratios as estimates of the relative risks for breast cancer death associated with the Recurrence Score, or a component of the Recurrence Score (univariate analyses). In addition, we estimated relative risks adjusted for tumor size and tumor grade (multivariate analyses). Model parameter estimation was done by maximum likelihood, and 95% Wald's confidence limits were calculated. Statistical significance was assessed via the likelihood ratio test [20]. In multivariate analyses, reported *P *values are for the addition of the given factor to a model including all other factors.

For women with ER-positive tumors, analyses were performed separately for those treated and not treated with tamoxifen. Because ER status was not a matching factor but was associated with our outcome, fewer than half of the ER-negative cases (*n *= 16) had matched controls who were also ER-negative. We therefore conducted conditional logistic regression analyses among those patients not treated with tamoxifen and generated relative risk estimates for ER-negative patients using terms for interaction with ER status. Finally, we conducted analyses of the full study population, using conditional logistic regression and interaction terms for ER status and tamoxifen therapy to obtain relative risk estimates for the different pre-specified patient groups characterized by these factors. However, because results were not materially different, we present only those results from analyses conducted within patient groups.

Methods developed by Langholz and Borgan [21] for nested case-control data were used to estimate the absolute risk of breast cancer death at 10 years and the corresponding 95% CIs. Estimates were calculated for subgroups of ER-positive patients, stratified on tamoxifen treatment and on Recurrence Score, tumor size, and tumor grade. In addition, 10-year risks for breast cancer death were calculated for subgroups of patients, when cross-classified by tumor size and grade and by Recurrence Score and tumor size and grade. The original Langholz and Borgan estimator assumed simple random sampling of controls from the set of known eligibles. In this study, we could not confirm eligibility without a review of the medical records, and therefore we sampled potentially eligible controls until up to three were confirmed for study inclusion. Thus, a slight modification to the original absolute risk estimator was necessary in order to reflect our sampling scheme (Additional file 1).

For comparison with the absolute risk estimates in the Kaiser population, the NSABP provided us with the Kaplan-Meier estimates for the probability of breast cancer death at 10 years among the 668 NSABP B-14 [12] tamoxifen-treated patients with node-negative breast cancer (previously published results included estimates for distant recurrence, relapse-free survival, and overall survival).

## Results

### Characteristics of cases and controls

Among 4,964 potentially eligible lymph node-negative invasive breast cancer patients, we identified 234 eligible cases and selected 631 controls with available tumor blocks. After loss of 7.9% of specimens because of insufficient tumor and 1% because of failed RT-PCR analysis, a total of 220 cases and 570 controls were available for statistical analyses. A total of 142 cases (64.6%) had three controls each, 66 cases (30.0%) had two controls each, and 12 cases (5.5%) had one control each. The distribution of factors available from the tumor registry (age, tumor size, race, diagnosis year, ER status, and tamoxifen treatment) among the evaluable cases and controls was generally similar to the distribution of these factors among the 239 potentially eligible cases and controls who were lost to the study. However, both lost cases and lost controls were slightly more likely than evaluable patients to be younger, to be not white, or to have smaller tumors.

Breast cancer deaths occurred a median of 4.9 years after diagnosis. Among the cases and controls, the median tumor size was 1.5 cm (range 0.2–7.0 cm). Cases and controls were similar with respect to matching factors including age, race, diagnosis year, and treatment with tamoxifen (Table 1). Nearly three-quarters of the study population was aged 50 years or older at diagnosis (median age 58 years) and about 80% were non-Hispanic white. Approximately two-thirds of patients were diagnosed with their breast cancer during the first 5 years of the accrual period (1985–1989). Overall, one-third of patients were treated with adjuvant tamoxifen. Before 1989, 11% of patients were treated with tamoxifen; from 1989 to 1994, 58% of patients were treated with tamoxifen. Among those treated with tamoxifen, the median duration was 4 years; approximately 10% had a year of treatment or less. Compared with controls, cases more commonly had tumors that were ER negative, larger, or more poorly differentiated. Cases were also more likely to have tumors with higher Recurrence Score values. Approximately 50% of patients had Recurrence Score values in the low risk category (that is, Recurrence Score <18).

For pre-specified analyses stratified by ER status and tamoxifen therapy, there were 55 cases and 150 matched controls who were tamoxifen treated and had ER-positive tumors according to RT-PCR assay. There were 110 cases and 251 matched controls who had ER-positive tumors according to RT-PCR assay and were not treated with tamoxifen. There were 16 cases with matched controls (*n *= 19) who had ER-negative tumors according to RT-PCR assay and were not treated with tamoxifen (out of a total of 52 ER-negative cases and 56 ER-negative controls). In addition, a small number of ER-negative patients were treated with tamoxifen (nine cases and three controls).

### Distribution of Recurrence Score risk categories by tumor size and tumor grade

The distributions of tumor size and/or tumor grade in patients categorized on the basis of the Recurrence Score as low risk (Recurrence Score <18), intermediate risk (Recurrence Score 18–30), or high risk (Recurrence Score ≥31) are shown in Table 2. The Recurrence Score was associated with tumor size and even more so with tumor grade. Nonetheless, a number of patients had large (>2 cm) and/or moderately or poorly differentiated tumors with low risk Recurrence Score values. In addition, a small percentage had small (≤1 cm) and/or well differentiated tumors with high risk Recurrence Score values. The concordance in the assessment of tumor grade between the two pathologists was moderate (kappa 0.53, 95% CI 0.49–0.59).

### Relative risks for breast cancer death: ER-positive patients

In ER-positive patients treated with tamoxifen, the risk of breast cancer death was positively and strongly associated with Recurrence Score when analyzed as a continuous variable, when categorized into quartiles, or when categorized based on pre-specified cutoff points (Table 3).

In ER-positive patients not treated with tamoxifen, the risk of breast cancer death was also positively associated with Recurrence Score (Table 3). As expected, the association of Recurrence Score with risk of breast cancer death appeared to be stronger among ER-positive patients treated with tamoxifen than among those not treated with tamoxifen.

Larger tumors and higher grade tumors were associated with an increased risk of breast cancer-specific mortality in both tamoxifen-treated and -untreated ER-positive patients (Table 4). When the Recurrence Score (continuous) was added to these multivariate models, it provided information on risk that was independent of tumor size and tumor grade. This was also true when the Recurrence Score was categorized into quartiles or when it was categorized based on pre-specified cutoff points (not shown). Note that even though there were differences in the assessment of grade between the two pathologists, similar to observations from other studies [22-26], the results were not materially different when tumor grade assessments from pathologist 2, rather than pathologist 1, were used (not shown).

**Figure 2:**
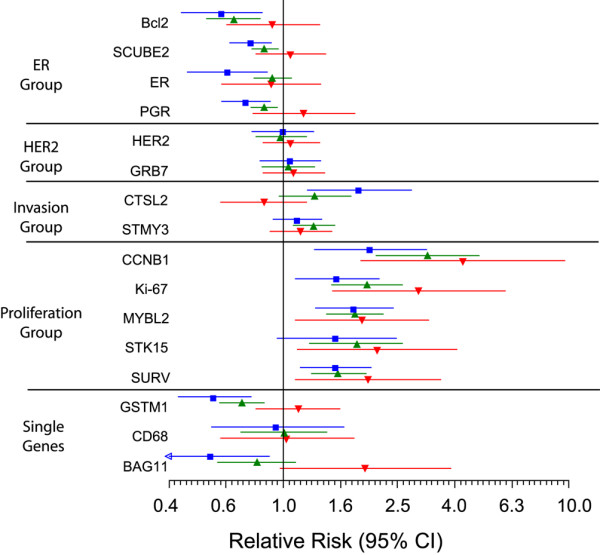
Relative risks (RRs) for death associated with expression of single genes. Findings are stratified by tamoxifen treatment and ER status. The position of each symbol indicates the RR. The length of the horizontal line through the symbol indicates the 95% CI. The blue boxes indicate the RRs and 95% CIs for ER-positive patients treated with tamoxifen, the green pyramids indicate the RRs and 95% CIs for ER-positive patients not treated with tamoxifen, and the red downward pointing triangles indicate the RRs and 95% CIs for ER-negative patients not treated with tamoxifen. CI, confidence interval; ER, estrogen receptor; RR, relative risk.

The relationships between the expression of the individual genes that comprise the Recurrence Score and risk of breast cancer death were generally similar in tamoxifen-treated and -untreated ER-positive patients (Figure 2). The risk for death was positively associated with expression of each of the five proliferation genes (cyclin B_1_, Ki-67, MYLBL2, STK15, and survivin). Positive associations were also observed for genes in the invasion group. The risk for death was negatively associated with expression of the ER-related genes (ER, PgR, BCL2, and SCUBE2). However, the associations between expression of the ER-related genes and risk were generally stronger for the tamoxifen-treated patients, especially with respect to the quantitative expression of the ER gene. Risk for death due to breast cancer was not statistically significantly associated with CD68, HER2, or GRB7 gene expression in patients treated or untreated with tamoxifen.

**Figure 3:**
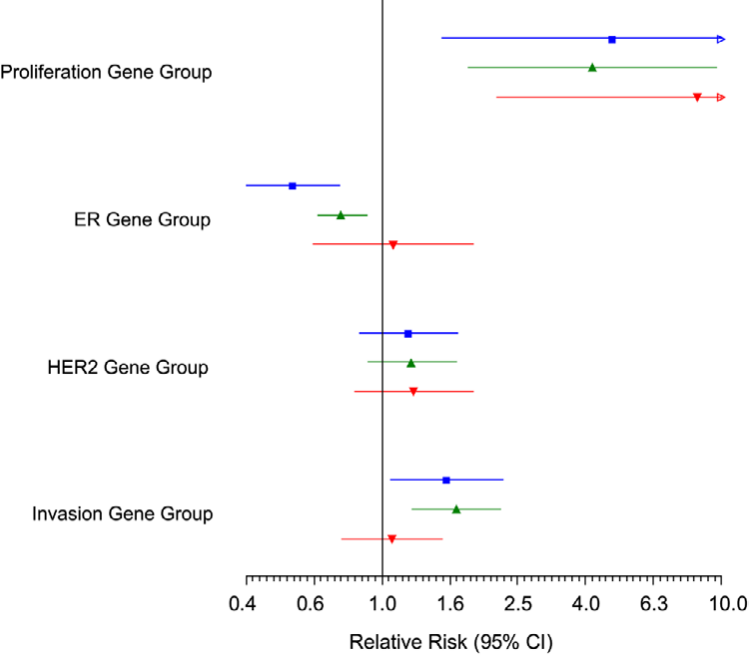
Relative risks (RRs) of death associated with gene group scores used in calculation of the Recurrence Score. Findings are stratified by tamoxifen treatment and ER status. The position of each symbol indicates the RR. The length of the horizontal line through the symbol indicates the 95% CI. The blue boxes indicate the RRs and 95% CIs for ER-positive patients treated with tamoxifen, the green pyramids indicate the RRs and 95% CIs for ER-positive patients not treated with tamoxifen, and the red downward pointing triangles indicate the RRs and 95% CIs for ER-negative patients not treated with tamoxifen. CI, confidence interval; ER, estrogen receptor; RR, relative risk.

The associations between expression of the gene group scores (as calculated in the Recurrence Score) and risk of breast cancer death were generally stronger than those for individual genes (Figure 3).

### Relative risks for breast cancer death: ER-negative patients

After adjusting for tumor size and tumor grade, the risk of breast cancer death was positively associated with the Recurrence Score (continuous variable in 50-unit increments) among ER-negative patients (relative risk 6.2, 95% CI 1.2–31.8). Risk for death was also strongly associated with the proliferation gene group (relative risk 5.1, 95% CI 2.0–13.5). For some individual genes, associations appeared to be more strongly positive in the ER-negative than in the ER-positive patients (that is, Cyclin B_1_, Ki-67, STK15, survivin). For a few genes, associations were in different directions for ER-negative and ER-positive patients (that is, BAG1, GSTM1, PgR). Note that estimates for ER-negative patients were often imprecise because of small numbers.

### Absolute risk of breast cancer death at 10 years: ER-positive patients

The risks for breast cancer death at 10 years for ER-positive patients treated with tamoxifen were 2.8% (95% CI 1.7–3.9%), 10.7% (95% CI 6.3–14.9%), and 15.5% (95% CI 7.6–22.8%) for patients with Recurrence Score values in the low, intermediate, and high risk categories, respectively (Table 5). Categories based on tumor size and/or grade identified a set of patients at similarly low risk of breast cancer death, although the Recurrence Score was able to identify a substantially larger subgroup of patients. For example, 63% of the controls were identified as low risk by the Recurrence Score, whereas only 31% of controls had a tumor less than 1 cm. Cross-classifying patients by Recurrence Score and tumor size and grade resulted in very imprecise estimates (that is, large CIs). Nonetheless, results suggest that among patients with Recurrence Score values categorized as low risk, tumor size and grade provide additional risk prediction information.

For ER-positive patients not treated with tamoxifen, the absolute risks for death at 10 years were higher than those for ER-positive patients treated with tamoxifen. The risks for breast cancer death were 6.2% (95% CI 4.5–7.9%), 17.8% (95% CI 11.8–23.3%), and 19.9% (95% CI 14.2–25.2%) for patients in the low, intermediate, and high risk Recurrence Score groups, respectively. Again, when cross-classifying patients by Recurrence Score and tumor size and grade, an improvement in risk prediction was suggested primarily for those with Recurrence Score values categorized as low risk.

## Discussion

In this large, population-based study of lymph node-negative patients not treated with chemotherapy, we found that the Recurrence Score was strongly associated with risk of breast cancer death among ER-positive patients treated with tamoxifen. We also found that the Recurrence Score was strongly associated with risk of breast cancer death among ER-positive patients not treated with tamoxifen and among ER-negative patients. In addition, we found that these associations remained after accounting for tumor size and grade, and that the Recurrence Score was able to identify a larger subset of patients with low risk of breast cancer death than was possible with either of these standard prognostic indicators.

Several limitations should be considered when interpreting our results. We lacked ER status from the medical record for a substantial proportion of patients, and we therefore classified ER status based on gene expression. However, the estimates of relative risk were not materially changed when analyses were restricted to the 84% of patients with ER status from the charts (data not shown). Because of the diagnosis years of the study, only approximately 30% of patients were treated with tamoxifen. Although this is consistent with what has been reported for other patient populations during this period [27], it limited the numbers of tamoxifen-treated patients for analysis. Given that the cases and controls were matched with respect to tamoxifen treatment, we could not directly examine whether the Recurrence Score is able to identify patients who are likely to respond to tamoxifen therapy. We did find a stronger association between the Recurrence Score and risk of breast cancer death among patients treated with tamoxifen than among those untreated with tamoxifen, suggesting that the Recurrence Score captures response to tamoxifen therapy as well as prognosis. This is most likely explained by the fact that the expression of ER-related genes was more strongly associated with breast cancer-specific mortality in the tamoxifen-treated patients than in those not treated with tamoxifen (Figure 2), and is also consistent with the established relationship between ER status of the tumor (by ligand binding or immunohistochemistry assay) and response to tamoxifen [4,28]. Our results are also consistent with findings from a study conducted among participants of the NSABP B-14 clinical trial, which randomized patients to tamoxifen versus placebo [29]. In this population, the tamoxifen benefit varied by Recurrence Score and was greatest for those with low Recurrence Score values. As expected, the strong association between the quantitative expression of the ER gene and tamoxifen benefit was largely responsible for this finding.

Currently, adjuvant hormonal and/or cytotoxic chemotherapy are recommended for most women with early-stage invasive breast cancer. Treatment decisions are based on axillary node status, age, tumor size, histologic tumor type, tumor grade, hormone receptor status (ER, PgR), and coexisting medical conditions [4]. Hormonal therapy is recommended for nearly all women with ER-positive tumors. Although tamoxifen is generally well tolerated, a significant proportion of women experience hot flashes and leg cramps, and up to 20% do not complete a 5-year course of tamoxifen therapy [30-32]. Despite its potential for serious adverse effects [33], cytotoxic chemotherapy has been recommended for most women with lymph node-positive disease and for node-negative patients with tumors greater than 1 cm or with unfavorable pathology [4-6]. Very little information is available to support the use of other clinical or biologic factors in selecting patients for adjuvant chemotherapy [4]. Most patients with node-negative disease who receive chemotherapy will not derive benefit, because they would not go on to have a recurrence even without such treatment. New prognostic and predictive tests are needed to better individualize therapy and confine systemic treatment, especially cytotoxic chemotherapy, to those patients who are most likely to benefit.

A growing number of studies suggest that multigene expression assays may be able to provide important prognostic and/or predictive information for breast cancer patients [34-42]. A variety of approaches and technologies are being used to select genes and characterize expression (for example, cDNA microarray chips, RT-PCR) in different types of pathology specimens (for example, fresh frozen tissue, formalin-fixed paraffin embedded tissue). No matter what the approach or technology, multiple, well conducted confirmatory studies using standardized methodologies will be needed before the clinical usefulness of any of these assays can be established.

This is the third study to evaluate the performance of the Recurrence Score among patients not treated with systemic chemotherapy [12,13]. The three studies used identical pre-specified scores and laboratory methods and were conducted among patients who were independent of those used for gene selection and Recurrence Score algorithm development. The results of the first study, conducted by Esteva and coworkers [13], differ substantially from our results and from those of the NSABP B-14 study [12]. In that study no association was found between the Recurrence Score and risk for distant recurrence among a series of 149 node-negative patients who were treated without adjuvant hormonal therapy or chemotherapy at the MD Anderson Cancer Center between 1978 and 1995, who had potentially 5 or more years of follow up, and for whom archived tissue was available. In contrast to the NSABP B-14 study, patients in the study conducted by Esteva and coworkers were not treated with tamoxifen. In contrast to our study, the outcome of interest was distant recurrence instead of breast cancer death. Although these may explain some of the differences observed, it is also possible that the study by Esteva and coworkers included a nonrepresentative group of patients. Patients with poorly differentiated tumors had better prognosis than those with well differentiated tumors, and there was a suggestion that patients with ER-negative tumors did better than those with ER-positive tumors.

Our relative risk estimates for the Recurrence Score in ER-positive, tamoxifen-treated patients are generally quite similar to those observed in the NSABP B-14 study. Relative risks associated with expression of individual genes were also very similar. In the NSABP B-14 study, the 10-year risk of breast cancer death was 3.1% (95% CI 1.2–5.0%) in the low risk group, 12.2% (95% CI 6.7–17.6%) in the intermediate risk group, and 27.0% (95% CI 20.4–33.6%) in the high risk group. Although very similar to our findings for the low and intermediate risk groups, the 10-year risk for the NSABP B-14 high risk group was higher than ours. Patients in the two studies had a comparable age distribution, and although those in the NSABP B-14 study were more likely to have larger tumors, their tumors were also more likely to be well differentiated. Therefore, there is uncertainty regarding the extent to which differences in the distribution of prognostic factors in the two study populations may explain the difference observed in absolute risk estimates for the high risk group. In the Kaiser population, tumor size and tumor grade remained statistically significantly associated with risk of breast cancer death in most multivariate models that also included the Recurrence Score, whereas only tumor grade remained independently associated with risk in the NSABP B-14 study. Tumor size was determined by pathology in the Kaiser study and by clinical examination in the NSABP B-14 study. Clinical examination of tumor size is generally less accurate and could have resulted in attenuated relative risk estimates.

## Conclusion

The Recurrence Score has now been shown to be strongly associated with risk of breast cancer-specific mortality among lymph node-negative, ER-positive, tamoxifen-treated patients participating in a clinical trial and among similar patients from the community setting. In both study populations, the Recurrence Score was able to identify a large subset of patients (approximately 50% or more) who were at very low risk of breast cancer death at 10 years. In both studies it was also observed that although the Recurrence Score was correlated with tumor size and grade, there were a number of patients with large and/or moderately or poorly differentiated tumors with low risk Recurrence Score values. Results from our study, and to a lesser extent the NSABP B-14 study, suggest that combining Recurrence Score, tumor grade, and tumor size provides better risk classification than any one of these factors alone. Other studies, either retrospective or prospective, will be needed to confirm our findings among lymph node-negative, ER-positive patients not treated with tamoxifen and among ER-negative patients. Two studies have been performed [43,44] and others are ongoing to assess the relationship between the Recurrence Score and the magnitude of chemotherapy benefit. Areas for future research also include the examination of whether the Recurrence Score assay provides prognostic or predictive information for patients treated with other hormonal agents or for patients with node-positive breast cancer, whether test performance can be further optimized by even more individualized dissection techniques, and whether the inclusion of additional genes or the inclusion of standard measures (tumor size, grade) may enhance risk prediction overall or for selected patient subgroups.

## Abbreviations

CI = confidence interval; ER = estrogen receptor; NSABP = National Surgical Adjuvant Breast and Bowel Project; PgR = progesterone receptor; RT-PCR = reverse transcriptase polymerase chain reaction.

## Competing interests

The following authors received support for study-related activities from Genomic Health, Inc., but have no other competing financial or nonfinancial interests: LAH, MJK, AC, NTB, DG, CPQ, BL, and LF. The remaining co-authors (SS, CA, MP, JB, MW, DW, and JH) are employees or consultants for Genomic Health, Inc.

## Authors' contributions

LAH participated in the design of the study, directed the data analysis, and drafted the manuscript. SS participated in the design of the study, in interpreting results, and in writing the manuscript. MKJ and AC performed statistical analyses and participated in interpreting results. NTB and CA participated in the development of the study methods and coordinated data collection. MP participated in the development of the study methods and in data collection. JB participated in the development of the study methods, in interpreting results, and in writing the manuscript. MW, DW, and JH participated in the design of the study and in interpreting results. DG participated in data collection and in interpreting results. CPQ participated in the study design, co-directed the data analysis, and participated in interpreting results. BL provided methods for estimating cumulative risks and participated in interpreting results. LF participated in interpreting results. All authors read and approved the final manuscript.

## Supplementary Material

Additional File 1 A Word document providing additional detail regarding the statistical analyses for estimating 10-year recurrence probabilities. Click here for file

## Figures and Tables

**Table 1:** Selected characteristics of the study population

Characteristic	Cases (*n *= 220)		Controls (*n *= 570)	
**Matched variables**				
Age at diagnosis (years)				
<40	17	(8%)	23	(4%)
40–49	42	(19%)	127	(22%)
50–59	64	(29%)	152	(27%)
60–74	97	(44%)	268	(47%)
Race/ethnicity				
White, non-Hispanic	172	(78%)	457	(80%)
White, Hispanic	7	(3%)	13	(2%)
Black	20	(9%)	47	(8%)
Asian	21	(10%)	53	(9%)
Surgery year				
1985–1989	146	(66%)	385	(68%)
1990–1994	74	(34%)	185	(32%)
Adjuvant tamoxifen				
No	156	(71%)	393	(69%)
Yes	64	(29%)	177	(31%)
**Unmatched variables**				
ER status from RT-PCR^1^				
Positive	168	(76%)	514	(90%)
Negative	52	(24%)	56	(10%)
Tumor size (cm)				
≤1	49	(22%)	193	(34%)
1.1–2	93	(42%)	255	(45%)
2.1–4	72	(33%)	114	(20%)
>4	6	(3%)	8	(1%)
Tumor grade (differentiation)^2^				
Well	25	(11%)	175	(31%)
Moderate	92	(42%)	261	(46%)
Poor	103	(47%)	134	(23%)
Recurrence Score				
Low risk (<18)	57	(26%)	322	(56%)
Intermediate risk (18–30)	54	(25%)	108	(19%)
High risk (≥31)	109	(50%)	140	(25%)

^1^Cutoff points based on RT-PCR values: ≤6.5 and >6.5 units. ^2^Bloom-Richardson grading criteria, pathologist 1. ER, estrogen receptor; RT-PCR, reverse transcription polymerase chain reaction.

**Table 2:** Distributions of tumor size and tumor grade in Recurrence Score categories for all 220 cases and 570 controls

Variable (number of patients)	Recurrence Score						
	< 18 (*n *= 379)		18–30 (*n *= 162)		≥30 (*n *= 249)		Correlation^1 ^ (*P *value)

Tumor size (cm)							0.25 (<0.0001)
≤1 (*n *= 242)	147	(61%)	51	(21%)	44	(18%)	
1.1–2 (*n *= 348)	173	(50%)	65	(19%)	110	(32%)	
2.1–4 (*n *= 186)	55	(30%)	44	(24%)	87	(47%)	
>4 (*n *= 14)	4	(29%)	2	(14%)	8	(57%)	
Tumor grade pathologist 1							0.54 (<0.0001)
Well (*n *= 200)	146	(73%)	44	(22%)	10	(5%)	
Moderate (*n *= 353)	199	(56%)	90	(25%)	64	(18%)	
Poor (*n *= 237)	34	(14%)	28	(12%)	175	(74%)	
Tumor size and grade							0.42 (<0.0001)
≤2 cm and well or ≤1 cm and moderate (*n *= 285)	204	(72%)	60	(21%)	21	(7%)	
>2 cm and well, or 1.1–2 cm and moderate, or ≤2 cm and poor (*n *= 330)	143	(41%)	63	(19%)	133	(40%)	
>2 cm and moderate/poor (*n *= 175)	41	(23%)	39	(22%)	95	(54%)	

^1^Spearman rank correlation

**Table 3:** Relative risks associated with recurrence score among ER-positive patients, stratified by treatment with tamoxifen

Score	Cases		Controls		RR^1^	(95% CI)	*P *value
Tamoxifen treated (55 cases and 150 controls)							
Recurrence Score							
Continuous^2^	55	(100%)	150	(100%)	7.6	(2.6–21.9)	<0.0001
Pre-specified categories							
Low risk (<18)	16	(29%)	95	(63%)	1.0	reference	
Intermediate risk (18–30)	22	(40%)	35	(23%)	4.0	(1.8–8.8)	
High risk (≥31)	17	(31%)	20	(13%)	6.2	(2.4–15.8)	
Quartiles^3^							0.0004
1st (0–8.25)	5	(9%)	38	(25%)	1.0	reference	
2nd (8.26–14.43)	6	(11%)	37	(25%)	1.0	(0.3–3.7)	
3rd (14.44–20.95)	15	(27%)	38	(25%)	2.9	(1.0–8.9)	
4th (20.96–78.78)	53	(53%)	37	(25%)	5.8	(2.0–16.6)	
**Tamoxifen untreated (110 cases and 251 controls)**							
Recurrence Score							
Continuous^2^	110	(100%)	251	(100%)	4.1	(2.1–8.1)	<0.0001
Pre-specified categories							<0.0001
Low risk (<18)	40	(36%)	160	(64%)	1.0	reference	
Intermediate risk (18–30)	32	(29%)	47	(19%)	2.7	(1.5–5.0)	
High risk (≥31)	38	(35%)	44	(18%)	3.3	(1.8–5.9)	
Quartiles^4^							<0.0001
1st (0–7.53)	11	(10%)	63	(25%)	1.0	reference	
2nd (7.54–14.25)	19	(17%)	64	(25%)	1.5	(0.7–3.4)	
3rd (14.26–21.86)	23	(28%)	62	(25%)	2.1	(0.9–4.9)	
4th (21.87–85.82)	57	(52%)	62	(25%)	5.3	(2.5-1.3)	

^1^Conditional logistic regression models include Recurrence Score variables only. ^2^Relative risks for Recurrence Score calculated with regard to an increment of 50 units (chosen to be consistent with and comparable with previous studies). ^3^Quartiles based on distribution among controls treated with tamoxifen. ^4^Quartiles based on distribution among controls not treated with tamoxifen. CI, confidence interval; ER, estrogen receptor; RR, relative risk.

**Table 4:** Relative risks of breast cancer death associated with tumor size, grade, and recurrence score among ER-positive patients, stratified by treatment with tamoxifen

Score	Cases		Controls		RR	95% CI	*P *value
**Tamoxifen treated (55 cases and 150 controls)**							
Multivariate analyses without Recurrence Score^1^							
Tumor size (cm)							0.009
Continuous^2^	55	(100%)	150	(100%)	2.6	(1.2–5.5)	
Grade (pathologist 1)							0.007
Well	6	(11%)	50	(33%)	1.0	reference	
Moderate	28	(51%)	69	(46%)	2.8	(1.1–7.4)	
Poor	21	(38%)	31	(21%)	4.5	(1.6–12.3)	
Multivariate analyses with Recurrence Score^3^							
Tumor size (cm)							0.013
Continuous^2^	55	(100%)	150	(100%)	2.5	(1.2–5.4)	
Grade (pathologist 1)							0.126
Well	6	(11%)	50	(33%)	1.0	reference	
Moderate	28	(51%)	69	(46%)	2.4	(0.9–6.4)	
Poor	21	(38%)	31	(21%)	2.8	(0.9–9.3)	
Recurrence Score							0.003
Continuous^4^	55	(100%)	150	(100%)	5.3	(1.6–17.2)	
**Tamoxifen untreated (110 cases and 251 controls)**							
Multivariate analyses without Recurrence Score^1^							
Tumor size (cm)							0.035
Continuous^2^	110	(100%)	251	(100%)	1.8	(1.0–3.1)	
Grade (pathologist 1)							<0.001
Well	17	(15%)	83	(33%)	1.0	reference	
Moderate	54	(49%)	131	(52%)	2.0	(1.1–3.6)	
Poor	39	(35%)	37	(15%)	4.7	(2.3–9.9)	
Multivariate analyses with Recurrence Score^3^							
Tumor size (cm)							0.036
Continuous^2^	110	(100%)	251	(100%)	1.8	(1.0–3.1)	
Grade (pathologist 1)							0.018
Well	17	(15%)	83	(33%)	1.0	reference	
Moderate	54	(49%)	131	(52%)	1.8	(1.0–3.4)	
Poor	39	(35%)	37	(15%)	3.2	(1.4–7.2)	
Recurrence Score							0.025
Continuous^4^	110	(100%)	251	(100%)	2.4	(1.1–5.2)	

^1^Conditional logistic regression models include tumor size and tumor grade only. ^2^Relative risks for tumor size calculated with regard to an increment of 2 cm. ^3^Conditional logistic regression models include tumor size, tumor grade, and Recurrence Score. ^4^Relative risks for Recurrence Score calculated with regard to an increment of 50 units (chosen to be consistent with and comparable to previous studies). CI, confidence interval; ER, estrogen receptor; RR, relative risk.

**Table 5:** Ten-year risk of death in relation to Recurrence Score and tumor size and grade among ER-positive patients, stratified by treatment with tamoxifen

Risk classifier	Cases	Controls	10-Year risk	
			
			%	95% CI
**Tamoxifen treated**				
Recurrence Score (55 cases and 150 controls)				
Low (<18)	29%	63%	2.8	(1.7–3.9)
Intermediate (18–30)	40%	23%	10.7	(6.3–14.9)
High (≥31)	31%	13%	15.5	(7.6–22.8)
Tumor size (55 cases and 150 controls)				
≤1 cm	16%	31%	3.6	(1.7–5.5)
1.1–2 cm	44%	43%	5.8	(3.7–7.8)
>2 cm	40%	26%	9.3	(5.5–13.1)
Tumor grade (55 cases and 150 controls)				
Well	11%	33%	2.1	(0.7–3.5)
Moderate	51%	46%	6.9	(4.7–9.0)
Poor	38%	21%	9.9	(6.0–13.7)
Tumor size and grade (55 cases and 150 controls)				
≤2 cm and well or ≤1 cm and moderate	15%	43%	2.5	(1.2–3.9)
>2 cm and well, or 1.1–2 cm and moderate, or ≤2 cm and poor	49%	39%	7.2	(4.9–9.5)
>2 cm and moderate/poor	36%	18%	11.5	(6.6–16.2)
Recurrence Score, tumor size and grade				
Recurrence Score low <18 (16 cases and 95 controls)				
≤2 cm and well or ≤1 cm and moderate	6%	46%	0.4	(0.0–1.0)
>2 cm and well, or 1.1–2 cm and moderate, or ≤2 cm and poor	63%	39%	4.1	(2.0–6.2)
>2 cm and moderate/poor	31%	15%	6.9	(1.8–11.7)
Recurrence Score intermediate 18–30 (22 cases and 35 controls)				
≤2 cm and well or ≤1 cm and moderate	32%	51%	6.8	(2.6–10.9)
>2 cm and well, or 1.1–2 cm and moderate, or ≤2 cm and poor	45%	29%	21.9	(5.0–35.8)
>2 cm and moderate/poor	23%	20%	13.5	(1.2–24.2)
Recurrence Score high ≥31 (17 cases and 20 controls)				
≤2 cm and well or ≤1 cm and moderate	0%	10%	12.2	(0.0–31.0)
>2 cm and well, or 1.1–2 cm and moderate, or ≤2 cm and poor	41%	60%	10.9	(2.9–18.3)
>2 cm and moderate/poor	59%	30%	28.9	(7.4–45.3)
**Tamoxifen untreated**				
Recurrence Score (110 cases and 251 controls)				
Low (<18)	36%	64%	6.2	(4.5–7.9)
Intermediate (18–30)	29%	19%	17.8	(11.8–23.3)
High (≥31)	35%	18%	19.9	(14.2–25.2)
Tumor size (110 cases and 251 controls)				
≤1 cm	29%	39%	8.5	(6.0–10.9)
1.1–2 cm	41%	45%	10.4	(7.8–12.9)
>2 cm	30%	16%	16.4	(11.4–21.0)
Tumor grade (110 cases and 251 controls)				
Well	15%	33%	5.5	(3.4–7.4)
Moderate	49%	52%	11.2	(8.6–13.8)
Poor	35%	15%	22.5	(15.9–28.6)
Tumor size and grade (110 cases and 251 controls)				
≤2 cm and well or ≤1 cm and moderate	27%	50%	6.2	(4.3–8.0)
>2 cm and well, or 1.1–2 cm and moderate, or ≤2 cm and poor	45%	37%	14.2	(10.8–17.4)
>2 cm and moderate/poor	27%	13%	19.1	(13.2–24.7)
Recurrence Score, tumor size and grade				
Recurrence Score low <18 (40 cases and 160 controls)				
≤2 cm and well or ≤1 cm and moderate	35%	63%	3.0	(1.7–4.3)
>2 cm and well, or 1.1–2 cm and moderate, or ≤2 cm and poor	48%	31%	11.0	(7.0–14.8)
>2 cm and moderate/poor	17%	6%	20.2	(6.1–32.2)
Recurrence Score intermediate 18–30 (32 cases and 47 controls)				
≤2 cm and well or ≤1 cm and moderate	34%	38%	17.0	(8.2–24.9)
>2 cm and well, or 1.1–2 cm and moderate, or ≤2 cm and poor	38%	34%	20.9	(10.2–30.4)
>2 cm and moderate/poor	28%	28%	22.0	(9.3–32.9)
Recurrence Score high ≥31 (38 cases and 44 controls)				
≤2 cm and well or ≤1 cm and moderate	13%	14%	25.0	(7.9–39.0)
>2 cm and well, or 1.1–2 cm and moderate, or ≤2 cm and poor	50%	59%	21.0	(13.8–27.5)
>2 cm and moderate/poor	37%	27%	25.3	(15.1–34.3)

CI, confidence interval; ER, estrogen receptor.
