# Supplementary material for: A population-based study of tumor gene expression and risk of breast cancer death among lymph node-negative patients
Source: Breast Cancer Res. 2006 May 31;8(3):R25. doi: 10.1186/bcr1412 (PMC1557737; doi:10.1186/bcr1412)
Supplement: Additional File 1 — A Word document providing additional detail regarding the statistical analyses for estimating 10-year recurrence probabilities. [file bcr1412-S1.doc]

**Statistical Appendix: Estimation of 10 Year Recurrence Probabilities**

Langholz and Borgan (1997) show how to estimate the risk of event occurrence for nested case-control studies that are a generalization of Kaplan-Meier and survival probability estimation for Cox regression. In an slight extension needed for the present study, the main building blocks for risk estimator is the baseline hazard “jumps” computed from each case-control set of the form

1/ ∑ w_j_ Y_j_ exp(Z_j_ β) (1)

where the sum is over all sampled subjects (including those who are not eligible controls for the case),Y_j_ indicates whether the subject is a eligible control, Z_j_ covariates (i.e., an indicator of recurrence score category, tumor size and grade), exp(Z_j_ β) is the estimated rate ratio, w_j_ is a “sampling weight” associated with subject j, and N-1 is the number of subjects identified as “potential controls,” subjects that possibly, but not necessarily are eligible controls for the given recurrence case. Suppose that S-1 potential controls are sequentially sampled until m-1 eligible controls are found (S includes the case plus the m-1 eligible controls plus the potential controls that were found be ineligible). This is exactly the “quota sampling” method of sampling described in Borgan, Goldstein, and Langholz (1995), p. 1760 with the weight for the m eligible subjects is given by

w_j = N *(m-1)/m * 1/(S-1).

Since only the eligible subjects contribute, (1) can be written as

1/ ∑ N*(m-1)/m * 1/(S-1) exp(Z_j_ β) (1)

where the sum is now only over eligible subjects*.* With this baseline hazard jump estimate, risk of recurrence was estimated using the “exact with no competing risks” method described in Langholz and Borgan (1997), for each given recurrence score and prognostic factor. The methods were implemented using the survival curve estimation procedure in SAS PHREG with log w_j specified as *offset*s in the model.

**References:**

Borgan O, Goldstein L, Langholz B. Methods for the analysis of sampled cohort data in the Cox proportional hazards model. Annals of Statistics. 1995;23:1749-1778.

Langholz B, Goldstein L. Fitting logistic models using conditional logistic regression when there are large strata. Computing Science and Statistics. 1997;29:551-555.
